# Four-Point Preprandial Self-Monitoring of Blood Glucose for the Assessment of Glycemic Control and Variability in Patients with Type 2 Diabetes Treated with Insulin and Vildagliptin

**DOI:** 10.1155/2015/484231

**Published:** 2015-10-26

**Authors:** Andrea Tura, Johan Farngren, Anja Schweizer, James E. Foley, Giovanni Pacini, Bo Ahrén

**Affiliations:** ^1^CNR Institute of Neuroscience, Corso Stati Uniti 4, 35127 Padova, Italy; ^2^Department of Clinical Sciences, Lund University, B11 BMC, 22184 Lund, Sweden; ^3^Novartis Pharma AG, 4002 Basel, Switzerland; ^4^Novartis Pharmaceuticals Corporation, One Health Plaza, East Hanover, NJ 07936, USA

## Abstract

The study explored the utility of four-point preprandial glucose self-monitoring to calculate several indices of glycemic control and variability in a study adding the DPP-4 inhibitor vildagliptin to ongoing insulin therapy. This analysis utilized data from a double-blind, randomized, placebo-controlled crossover study in 29 patients with type 2 diabetes treated with vildagliptin or placebo on top of stable insulin dose. During two 4-week treatment periods, self-monitoring of plasma glucose was undertaken at 4 occasions every day. Glucose values were used to assess several indices of glycemic control quality, such as glucose mean, GRADE, M-VALUE, hypoglycemia and hyperglycemia index, and indices of glycemic variability, such as standard deviation, CONGA, J-INDEX, and MAGE. We found that vildagliptin improved the glycemic condition compared to placebo: mean glycemic levels, and both GRADE and M-VALUE, were reduced by vildagliptin (*P* < 0.01). Indices also showed that vildagliptin reduced glycemia without increasing the risk for hypoglycemia. Almost all indices of glycemic variability showed an improvement of the glycemic condition with vildagliptin (*P* < 0.02), though more marked differences were shown by the more complex indices. In conclusion, the study shows that four-sample preprandial glucose self-monitoring is sufficient to yield information on the vildagliptin effects on glycemic control and variability.

## 1. Introduction

The combination of dipeptidyl peptidase-4 (DPP-4) inhibition with insulin is a glucose lowering strategy for the treatment of type 2 diabetes which has gained considerable interest during recent years [1]. The two treatments have synergistic mechanistic actions [1, 2] and the results of clinical trials show that the combination of DPP-4 inhibition and insulin improves glycemia with low risk of hypoglycemia and prevention of weight gain [3–8]. Whether the combination of DPP-4 inhibition and insulin also minimizes glycemic variability is not completely known but would be important because glycemic variability may contribute to the long-term risk in type 2 diabetes [9–12]. Previous studies on DPP-4 inhibition and glycemic variability typically analyzed the combination with metformin or sulfonylureas [13–18], and not the combination with insulin, when the hypoglycemic drive is stronger. To our knowledge, few studies analyzed glycemic variability in patients under DPP-4 inhibition and insulin, and the study duration was limited to few days [19, 20]. More importantly, however, previous studies on DPP-4 inhibition and glycemic variability assessed only few, basic indices of glycemic variability (typically, the standard deviation of the glycemic values), which only partially and incompletely characterize the glycemic variability.

The aim of this study was to explore the utility of using only 4 preprandial readings per day of blood glucose for the estimation of various indices of glycemic control quality and variability, in order to examine whether with this approach it is possible to verify the improved glycemic variability by DPP-4 inhibition (vildagliptin) in combination with insulin. In a recent study we demonstrated that vildagliptin in combination with insulin reduced glucagon levels at hyperglycemia and sustained the glucagon counterregulation to hypoglycemia, which yielded lower glucose levels at hyperglycemia and prevention of hypoglycemia in type 2 diabetes [21]. In this study, we focused on the characterization of glycemic control quality and variability, and we also assessed the relation between these indices and the glycosylated haemoglobin [22]. Of note, it may be clinically relevant to show that few preprandial glucose readings per day are sufficient to yield information about the ability of vildagliptin to improve the glycemic condition, that is, the levels of both glycemic control and variability. In fact, patients with type 2 diabetes under insulin therapy typically measure glycemia preprandially, before insulin administration, and possibly at bedtime. Thus, it may be important to show that some information on the glycemic control and variability can be obtained with such readings only, without the need of further readings that are usually not taken in the clinical practice.

## 2. Materials and Methods

### 2.1. Subjects

This study utilized data from a single-center (Lund University), double-blind, randomized, placebo-controlled crossover study (NCT01219400) in patients with type 2 diabetes who were treated with a stable dose of insulin (long-acting insulin (34 ± 16 U) with (*n* = 22) or without (*n* = 7) short-acting insulin (21 ± 13 U)), and with or without oral hypoglycemic agents, with the primary end point to examine the glucagon response to insulin-induced hypoglycemia in these patients [21]. Eligible patients were 18 years or older with a diagnosed antibody-negative type 2 diabetes, a glycosylated haemoglobin (HbA1c) value between 6.5 and 8.5% (48–68 mmol/mol), and fasting plasma glucose levels (FPG) <15 mmol/L. Patients were excluded if they were pregnant or lactating, had type 1 diabetes, had acute infection during the 4 weeks preceding the study, had severe hypoglycemia within 2 weeks before the study (i.e., hypoglycemia which needed assistance of another person), had severe liver disease, were blood donors, or were treated with growth hormone or an oral steroid during the 2 months preceding the study. Twenty-nine patients were eventually enrolled in the study (age = 57.9 ± 1.2 years; BMI = 30.8 ± 0.8 kg/m^2^; FPG = 8.6 ± 0.4 mmol/L, HbA1c = 59.9 ± 1.5 mmol/mol, diabetes duration = 14.6 ± 1.6 years). The daily insulin dose was not significantly changed during the treatment periods.

### 2.2. Study Procedure

Each patient attended a screening visit at week −4, during which inclusion/exclusion criteria were assessed. Eligible patients were randomized at visit 2 (day 1) and were expected to complete two treatment periods, receiving a different blinded study medication during each period (Vildagliptin, Novartis Pharma AG, Basel, Switzerland, 50 mg twice a day, or placebo, in random order, randomized in blocks by the university hospital pharmacist). At the baseline (day 1) visit, the study medication was dispensed for 4 weeks for outpatient treatment. Study medication was then discontinued, and a 4-week washout period occurred before the alternative 4-week treatment period. After each 4-week study period, a hypoglycemia clamp was undertaken to evaluate glucagon secretion; these results have been previously reported [21]. During the two 4-week study periods, self-monitoring of plasma glucose was undertaken at 4 occasions every day (before breakfast, before lunch, before dinner, and at bedtime). Samples were taken through a finger and measured by an AccuCheck glucometer (Roche, Basel, Switzerland). HbA1c was also taken before and after the respective 4-week periods. The protocol was approved by the Ethics Committee of Lund University and the Swedish Medical Product Agency, and all patients gave written informed consent before entering the study. The study was conducted using good clinical practice and in accordance with the Declaration of Helsinki.

### 2.3. Calculations

For a detailed analysis of the glucose data, we assessed several indices of glycemic control quality and glycemic variability. The indices of glycemic control quality assess to what extent the glucose data remain near a target value or in a target range. There are both basic indices of descriptive statistics and more complex indices. Descriptive indices include glucose mean, maximum, minimum, 50th percentile (median), percentage of glucose values in a target range (3.9–11.1 mmol/L), and below and above a target value (3.9 and 11.1 mmol/L, resp.). It should be noted that 3.9 and 11.1 mmol/L are arbitrary thresholds, although assumed in many studies [23]. The more complex indices are as follows:

(i) GRADE (Glycemic Risk Assessment Diabetes Equation) [24]: glucose values are transformed to yield a continuous curvilinear response with a minimum at 5.0 mmol/L and high adverse weighting to hyperglycemia and hypoglycemia: GRADE = 425 × {log_10_⁡[log_10_⁡(Gluc_*n*_)] + 0.16}^2^, with the glucose value, Gluc_*n*_, in mmol/L; then, average value is taken.
(ii) M-VALUE (not to be confused with the insulin sensitivity index from the clamp) [25]: it is a weighted average of the glucose values, with progressively larger penalties for more extreme values, thus resulting in higher values of the index: M-VALUE = |10 × log_10_⁡Gluc_*n*_/IGV|^3^, where IGV is the ideal glucose value, typically assumed, as in this study, to be equal to 6.7 mmol/L; again, average value is then taken.
(iii) Hypoglycemia index [23] is the average of hypoglycemic values; if blood glucose value is lower than a given threshold, the formula for the index is Hypo index = (LLTR − Gluc_*n*_)^2.0^/30, with Gluc_*n*_ and threshold, LLTR (lower limit of target range), in mg/dL (typically, LLTR = 4.4 mmol/L).
(iv) Hyperglycemia index [23] is the average of hyperglycemic values; if blood glucose value is higher than a given threshold, the formula for the index is Hyper_index = (Gluc_*n*_ − ULTR)^1.1^/30, with Gluc_*n*_ and threshold, ULTR (upper limit of target range), in mg/dL (typically, ULTR = 7.8 mmol/L).
(v) IGC (Index of Glycemic Control) [23] is the sum of hyperglycemia index and hypoglycemia index.
(vi) LBGI (Low Blood Glucose Index) [26]: it consists in a transformation that normalizes the blood glucose scale: LBGI = 1.509 × {[log_*e*_⁡(Gluc_*n*_)]^1.084^ − 5.381}, for blood glucose values less than 6.2 mmol/L; then, a risk value is assigned to each blood glucose reading as follows: Risk(LBGI) = 10 × LBGI^2^; finally, average value is taken.
(vii) HBGI (High Blood Glucose Index) [26]: similarly to LBGI, it consists in a transformation that normalizes the blood glucose scale, for blood glucose values higher than 6.2 mmol/L: the expression of HBGI is the same as for LBGI;
(viii) ADRR (Average Daily Risk Range) [27]: it is the sum of LBGI and HBGI, calculated with the minimum and the maximum glucose value, respectively.

The indices of glycemic variability measure to what extent data oscillate: the higher the variability, the higher the value of such indices. Some basic indices of this type are the glucose standard deviation (SD) and the interquartile range. More complex indices are as follows:

(i) CONGA (Continuous Overlapping Net Glycemic Action) [28]: it is the SD of the difference between values obtained exactly *n* minutes apart; typically, *n* is equal to 60 min (or its multiples), but in this case we performed the analysis over the glucose data available, despite the fact that the time interval between consecutive values was typically higher than one hour.
(ii) J-INDEX [23] is a combination of information from mean and SD of all glucose values: J-INDEX = 0.001 × (mean + SD)^2^.
(iii) MAGE (Mean Amplitude of Glycemic Excursion) [29] is the arithmetic mean of the glycemic excursions that are greater than one SD; MAGE (pos.) and MAGE (neg.) consider in particular the positive and the negative excursions, respectively.

### 2.4. Statistical Analysis

After testing for normality of indices values distributions, possible differences in the values of the indices after vildagliptin and after placebo were assessed by nonparametric Wilcoxon Signed Rank Test. *P* < 0.05 was considered statistically significant. Possible relationships between variables were assessed by linear regression analysis. Values are reported as mean ± SE.

## 3. Results

**Figure 1:**
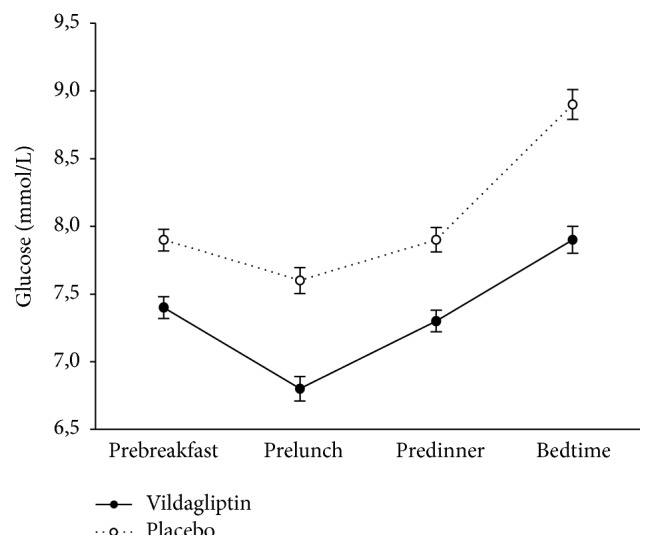
Mean average glucose data before breakfast, before lunch, before dinner, and at bedtime in patients with type 2 diabetes treated with vildagliptin (solid line) or placebo (dotted line) as add-on to insulin in a crossover design (*n* = 29). Data are mean ± SE.

The average glucose pattern during the day (before breakfast, before meal, before dinner, and at bedtime) with vildagliptin and with placebo when compiled together for all days is reported in Figure 1. It is seen that vildagliptin reduced glucose levels throughout the day. This was accompanied by a reduction in HbA1c with vildagliptin by −0.5 ± 0.1% (−4.7 ± 0.6 mmol/mol; *P* < 0.001) whereas, during treatment with placebo, there was no significant change in HbA1c (change by −0.1 ± 0.1% [−1.1 ± 0.6 mmol/mol]).

The values of the indices of both glycemic control quality and glycemic variability, as defined earlier, are reported in Table 1. Vildagliptin improved the glycemic condition compared to placebo. Mean (and median) and maximum glycemic levels were reduced with vildagliptin, whereas the minimum value, typical of hypoglycemia, remained unchanged. Vildagliptin also reduced the percentage of values over 11.1 mmol/L (and this is also confirmed by the hyperglycemia index), whereas the percentage of values in the target range (3.9–11.1 mmol/L) appears to be not significantly changed. As regards the low glycemic values (<3.9 mmol/L), vildagliptin and placebo showed again similar behavior. Furthermore vildagliptin tended to reduce the hypoglycemia index (lower value compared to placebo), though statistical significance was not reached. GRADE and M-VALUE showed that vildagliptin increased the glucose values near the respective target value, that is, 5.0 and 6.7 mmol/L (again, lower values compared to placebo); the higher value of LBGI and the lower value of HBGI consistently confirmed the glucose lowering effect of vildagliptin, also further confirmed by ADRR. The indices of glycemic variability consistently showed an improvement of the glycemic condition with vildagliptin, with values that were all significantly reduced, except for the interquartile range, where the difference did not reach statistical significance.

**Figure 2:**
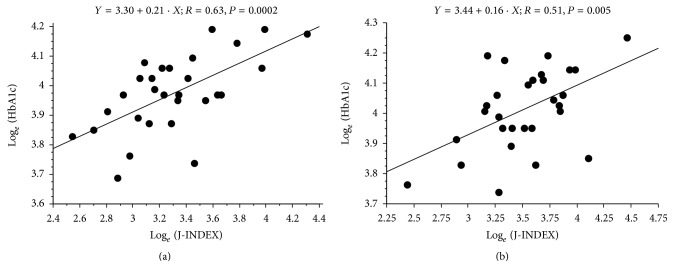
Relationships between an index of glycemic variability (J-INDEX) and glycated hemoglobin after treatment, in the case of vildagliptin (a) and placebo (b). Variables were logarithmically transformed.

Both with vildagliptin and placebo, there was a weak but significant relationship between HbA1c after treatment and glucose mean during the 4-week treatment (*R* = 0.57, *P* = 0.001, and *R* = 0.52, *P* = 0.004, resp.). Significant relationships with HbA1c after treatment were also found for glucose median, GRADE, M-VALUE, hyperglycemia index, and HBGI, with typically stronger relationship in vildagliptin (*R* = 0.47–0.61, *P* = 0.01–0.0004 for vildagliptin, *R* = 0.42–0.56, *P* = 0.02–0.002 for placebo, resp.). Percentage in target index showed a significant inverse relationship with HbA1c after treatment (*R* = 0.50, *P* = 0.006 for vildagliptin, *R* = 0.51, *P* = 0.004 for placebo). We found also that every index of glycemic variability was related to HbA1c after treatment (*R* between 0.49 and 0.63, *P* < 0.007 with vildagliptin, and *R* between 0.39 and 0.51, *P* < 0.038 with placebo; see Figure 2).

## 4. Discussion

In this study, we show that glycemic control and variability as estimated by several indices on 4-point preprandial self-monitoring of blood glucose (SMBG) is improved during the course of the treatment with vildagliptin add-on to insulin therapy. In fact, the great majority of indices showed a significant amelioration with vildagliptin compared to placebo. In particular, the indices showed that vildagliptin reduced glycemia without increasing the risk for hypoglycemia, which is a clinical experience with vildagliptin added to insulin [2, 3]. In fact, the minimum glucose value was not different between vildagliptin and placebo, suggesting reduction of glucose without increased hypoglycemia risk. This conclusion is strengthened by the hypoglycemia index, which was again not different between the groups. The improvement of glycemic variability by vildagliptin was probably related to its mechanism of action: to reduce glucagon at hyperglycemia and sustain glucagon counterregulation [2, 21]. Of note, some indices, both for glycemic control and glycemic variability, would remain significantly improved with vildagliptin even after the conservative Bonferroni correction for multiple statistical comparisons.

In previous studies, it has been shown that DPP-4 inhibitors improve glycemic variability when added to metformin, although only few indices (typically SD) were evaluated [13–18]. In the study by Mori et al., it was shown that adding sitagliptin to insulin narrowed the range of 24 h glucose fluctuations [19]. We confirm here that these indices were reduced by vildagliptin when added to insulin. Strength of this study was the crossover design and the use of several indices which better characterize the glucose patterns even with only 4 points. Of note, previous studies showed that glycemic variability can in fact be reliably assessed by SMBG, even with few points per day [30, 31]. Specifically, in the study [30], an average of 4.84 samples/day was collected (range 3–7), whereas in the study [31] 70 samples were taken over a period of 4 weeks, thus in line with the number of samples of our study (112 samples over the 4 weeks period).

The assessed indices were grouped into two different categories, that is, indices of glycemic control quality and indices of glycemic variability, although for some indices such division may be arbitrary and some indices may belong to both categories. In fact, for a detailed analysis of repeated glucose data, it is important to evaluate a sufficiently wide battery of indices of both glycemic control quality and glycemic variability. In a given population, some of these indices may show similar results; thus the calculation of all of them may be redundant, but this is not always the case. In fact, many of the indices, which were evaluated in this study, have specificities that may provide peculiar information, since each index weighs the different glucose values differently. For example, one index may disclose subtle differences in the glycemic condition between two populations that are not disclosed by other indices. In fact, Rodbard claimed that different indices may have different abilities to detect responses to therapeutic interventions [23]. In more detail, regarding the indices of glycemic control quality, some indices predominantly assess low glucose values (glucose minimum, percentage below target, hypoglycemia index, and LBGI), whereas other indices predominantly assess high glucose values (glucose maximum, percentage above target, hyperglycemia index, and HBGI). Furthermore, other indices focus on euglycemia (percentage in target) and, finally, other indices combine these three facets of the glycemic condition (glucose mean and median, GRADE, M-VALUE, IGC, and ADRR). Among the indices addressing a specific aspect of glycemia, there are some relevant differences: as an example, the M-VALUE, with the ideal glucose value, IGV, equal to 6.7 mmol/L (as used in this study) assigns equal penalty (i.e., the index assumes the same value) for 3.3 mmol/L and 13.3 mmol/L; as regards GRADE, it assigns equal penalty to 2.8 mmol/L and 11.9 mmol/L. Even the indices of glycemic variability do not always provide the same results. For instance, it is claimed that J-INDEX may be relatively insensitive to variations in the low glucose range [23].

It should be noted that the percentage in target index did not reach statistically significant difference between adding vildagliptin or placebo to ongoing insulin therapy. This may be due to the lack of postprandial glucose readings. However, though the percentage in target index was not significantly different between vildagliptin and placebo, all the other indices (except IGC) were significantly different, in some cases also displaying very low *P* values (such as GRADE: see Table 1). This suggests the importance of assessing several indices, and especially those more complex, for a complete evaluation of the glycemic condition. The importance of assessing several indices is also confirmed by some results of the glycemic variability indices: in fact, the simple, basic interquartile range index failed to display a significant difference between vildagliptin and placebo, whereas all the other indices were significantly different. Moreover, it should be noted that more markedly significant differences were displayed by the more complex indices (J-INDEX, CONGA, and MAGE), thus underlying the potential advantages of such indices compared to the simplest indices. Of note, in this study, the glycemic variability index showing the most marked difference between vildagliptin and placebo was J-INDEX, despite the fact that in previous studies it was reported to be relatively insensitive [23]. This further suggests that the use of a wide battery of indices may be beneficial for accurate and reliable analyses of the glycemic patterns.

We also assessed possible relationships between HbA1c and glucose values. In fact, it is known that in type 2 diabetes HbA1c is related to glucose values, even when glucose values are self-monitored [32]. We found that there was a significant relationship between HbA1c after treatment and the glucose mean of the 4-week period, though the relationship was relatively weak. Possible explanation is that our glucose values were referred to a period of 4 weeks only, whereas it is well known that HbA1c is related to glucose values of longer periods. Furthermore, since it has been postulated that glycemic variability may contribute to glycation [33], we also assessed possible relationships between HbA1c and glycemic variability indices. We found that the relationship exists with any index, both with vildagliptin and placebo; yet it appeared that the relationships were stronger with vildagliptin than with placebo.

In this study, we aimed to show that few, preprandial glycemic readings are adequate to assess the glycemic control and variability in patients with type 2 diabetes, which were under insulin and vildagliptin treatment. In fact, the used protocol has some advantages: first, it allowed the assessment of the specific effect of vildagliptin on the fasting, or at least preprandial, glycemic condition; furthermore, such protocol limited the discomfort to the patients, requiring few measurements per day, which are already typically taken in such kind of patients. Thus, we showed that even few glucose samples, not requiring specific medical instrumentation (such as devices for continuous glucose monitoring), or additional self-monitoring glycemic readings (compared to those typically performed in insulin treated patients with type 2 diabetes), are sufficient to provide relevant information on glycemic control and variability: in fact, improvements in glycemic control and variability due to vildagliptin can be observed even when postprandial excursions are not determined. Of course, including postprandial readings may show even more marked differences in glycemic control or variability due to vildagliptin, but in this study our priority was detecting significant vildagliptin effects with minimal requirements in terms of glycemic readings, for easier applicability in the clinical practice. It should also be noted that, similarly to our study, previous studies were in fact focused on the assessment of glycemic variability limited to fasting values [34–38].

## 5. Conclusions

With the assessment of several indices of glycemic control quality and glycemic variability, we comprehensively evaluated the glycemic condition of patients with type 2 diabetes that underwent vildagliptin treatment in addition to insulin, which is a combination only partially investigated in previous studies. The study showed that there is a clear improvement of both glycemic control quality and glycemic variability after only 4 weeks of vildagliptin therapy.

The study underlines that it is important to compute several indices to evaluate daily glucose levels and fluctuations, since not all indices have similar ability to show possible differences in the glycemic control and variability condition.

Furthermore, the study showed that a simple protocol, requiring few self-monitoring preprandial readings, is sufficient to disclose some significant effects of vildagliptin on glycemic control and variability. It will be of interest to apply similar methodologies to future comparative studies involving two active drugs in combination with insulin.

## Figures and Tables

**Table 1:** Indices of glycemic control quality and glycemic variability in patients with type 2 diabetes after placebo and after vildagliptin (data are mean ± SE). For the explanation of the various indices see the Methods section.

	Placebo	Vildagliptin	*P* value
Indices of glycemic control quality			
Mean (mmol/L)	8.11 ± 0.25	7.36 ± 0.23	<0.0001
Maximum (mmol/L)	16.37 ± 1.80	13.54 ± 0.66	0.007
Minimum (mmol/L)	3.76 ± 0.20	3.66 ± 0.19	0.37
50th percentile (median) (mmol/L)	7.82 ± 0.25	7.13 ± 0.24	0.0001
Percentage below target (3.9 mmol/L) (%)	2.0 ± 0.6	3.1 ± 0.9	0.055
Percentage in target (3.9–11.1 mmol/L) (%)	86.1 ± 2.4	89.8 ± 2.2	0.062
Percentage above target (11.1 mmol/L) (%)	11.9 ± 2.3	7.0 ± 2.0	0.002
GRADE (unitless)	6.61 ± 0.59	5.17 ± 0.52	<0.0001
M-VALUE (unitless)	6.22 ± 1.18	4.97 ± 0.95	0.004
Hypoglycemia index (unitless)	7.67 ± 1.45	5.66 ± 1.07	0.093
Hyperglycemia index (unitless)	1.97 ± 0.20	1.61 ± 0.20	0.002
IGC (unitless)	9.81 ± 1.32	7.32 ± 1.04	0.059
LBGI (unitless)	0.64 ± 0.15	1.03 ± 0.20	0.002
HBGI (unitless)	4.57 ± 0.66	3.12 ± 0.56	<0.0001
ADRR (unitless)	45.0 ± 7.3	35.5 ± 3.8	0.043

Indices of glycemic variability			
Standard deviation (mmol/L)	2.27 ± 0.21	2.00 ± 0.17	0.015
Interquartile range (mmol/L)	2.87 ± 0.24	2.68 ± 0.26	0.092
CONGA (mmol/L)	3.12 ± 0.29	2.71 ± 0.22	0.011
J-INDEX (10^−3^ (mmol/L)^2^)	0.12 ± 0.01	0.09 ± 0.01	<0.0001
MAGE (mmol/L)	4.80 ± 0.48	4.16 ± 0.33	0.010
MAGE (pos.) (mmol/L)	4.82 ± 0.48	4.20 ± 0.32	0.020
MAGE (neg.) (mmol/L)	4.77 ± 0.47	4.13 ± 0.35	0.007
